# Adaptive divergence in a scleractinian coral: physiological adaptation of *Seriatopora hystrix *to shallow and deep reef habitats

**DOI:** 10.1186/1471-2148-11-303

**Published:** 2011-10-17

**Authors:** Pim Bongaerts, Cynthia Riginos, Kyra B Hay, Madeleine JH van Oppen, Ove Hoegh-Guldberg, Sophie Dove

**Affiliations:** 1 School of Biological Sciences, The University of Queensland, St Lucia, QLD 4072, Australia; 2 ARC Centre of Excellence for Coral Reef Studies, The University of Queensland, St Lucia, QLD 4072, Australia; 3 Heron Island Research Station, The University of Queensland, Heron Island, QLD 4680, Australia; 4 Australian Institute of Marine Science, Townsville, QLD 4810, Australia; 5 Global Change Institute, The University of Queensland, St Lucia, QLD 4072, Australia

## Abstract

**Background:**

Divergent natural selection across environmental gradients has been acknowledged as a major driver of population and species divergence, however its role in the diversification of scleractinian corals remains poorly understood. Recently, it was demonstrated that the brooding coral *Seriatopora hystrix *and its algal endosymbionts (*Symbiodinium*) are genetically partitioned across reef environments (0-30 m) on the far northern Great Barrier Reef. Here, we explore the potential mechanisms underlying this differentiation and assess the stability of host-symbiont associations through a reciprocal transplantation experiment across habitats ('*Back Reef*', '*Upper Slope*' and '*Deep Slope*'), in combination with molecular (mtDNA and ITS2-DGGE) and photo-physiological analyses (respirometry and HPLC).

**Results:**

The highest survival rates were observed for native transplants (measured 14 months after transplantation), indicating differential selective pressures between habitats. Host-symbiont assemblages remained stable during the experimental duration, demonstrating that the ability to "shuffle" or "switch" symbionts is restricted in *S. hystrix*. Photo-physiological differences were observed between transplants originating from the shallow and deep habitats, with indirect evidence of an increased heterotrophic capacity in native deep-water transplants (from the '*Deep Slope*' habitat). Similar photo-acclimatisation potential was observed between transplants originating from the two shallow habitats ('*Back Reef*' and '*Upper Slope*'), highlighting that their genetic segregation over depth may be due to other, non-photo-physiological traits under selection.

**Conclusions:**

This study confirms that the observed habitat partitioning of *S. hystrix *(and associated *Symbiodinium*) is reflective of adaptive divergence along a depth gradient. Gene flow appears to be reduced due to divergent selection, highlighting the potential role of ecological mechanisms, in addition to physical dispersal barriers, in the diversification of scleractinian corals and their associated *Symbiodinium*.

## Background

Coral reefs are among the most diverse ecosystems on the planet, second only to tropical rainforests in the number of species they harbour [1]. Although the mechanisms that regulate and sustain diversity in both tropical rainforests and coral reefs remain heavily debated, it is clear that the characteristic environmental heterogeneity of these ecosystems must play an integral role by providing important axes for niche diversification [6,7]. In particular, the steep environmental gradients encountered on coral reefs [8] should exert strong differential selective pressures on coral populations and lead to local adaptation at environmental extremes [9]. Yet, direct evidence for the occurrence of such local adaptation across environmental gradients in scleractinian corals remains limited. Given the sedentary nature of corals and increasing evidence for predominantly localised dispersal [10], however, local adaptation may be common [9,11] and potentially play an important role in the genetic and phenotypic diversification of scleractinian corals and their associated *Symbiodinium*.

Ecological adaptation through divergent selection can contribute to genetic divergence of populations (i.e., adaptive divergence), when the strength of selection overcomes the homogenizing effect of gene flow and recombination [12]. Although genetic divergence is often the consequence of reductions in gene flow associated with physical barriers (e.g., geographic isolation), adaptive divergence can also reduce gene flow through the establishment of ecological reproductive barriers [13]. Rather than post-zygotic reproductive barriers, such as intrinsic hybrid sterility and genomic incompatibilities, gene flow between populations can be hampered through pleiotropic effects of selection [14]. For example, selection against migrants from opposing parental environments (i.e., immigrant inviability) can reduce the chance of heterospecific mating encounters, and as such can represent an ecologically-based pre-zygotic barrier to gene flow [15]. Similarly, divergent selection is expected to result in selection against hybrids or intermediate phenotypes that are maladapted to either parental habitat, contributing to post-zygotic reproductive barriers [16]. The evolution of reproductive isolation through divergent selection has been relatively well-studied in both laboratory and natural settings [17-19]. However, it is unclear to what extent ecologically-based divergent selection, which should be ubiquitous on coral reefs given their environmental heterogeneity, contributes to the establishment of reproductive barriers in scleractinian corals.

Ecological barriers to gene flow can ultimately lead to complete reproductive isolation and the formation of new species, i.e., ecological speciation (reviewed in [14]). Although ecological barriers can be important for speciation under different rates of migration [15] and in various geographical contexts (e.g., allopatric or sympatric) [20], much attention has been given to how ecological selection can lead to reproductive isolation in the absence of physical barriers to gene flow (i.e., in sympatry). As migration rates are expected to be initially very high in sympatry, selection must be strong enough to overwhelm migration during the early stages of divergence. In later stages, migration may be reduced through ecological effects of selection resulting in a positive feedback loop of accelerating divergence [15]. Coyne and Orr [19] argue that sympatric speciation can be inferred when the following expectations are met: (1) lineages exhibit a present-day sympatric distribution, (2) lineages form a monophyletic cluster of sister species that are (3) reproductively isolated, and (4) that an allopatric contribution is unlikely. However, there are many caveats associated with these criteria, and the inability to satisfy all these criteria has greatly hampered progress towards understanding the importance of ecological speciation in the marine realm. For example, under these criteria, sympatric speciation can only be detected if there are no major changes in geographic distributions and/or subsequent allopatric divergence after the initial sympatric speciation [21]. Additionally, the last criterion poses allopatric speciation as the null model, which effectively limits the verification of sympatric speciation to unique isolated terrestrial and freshwater settings (e.g., [22,23]) where any contribution to historic allopatry can be confidently excluded. Overall, the criteria impose an artificial binary categorisation of the geographic context (allopatric or sympatric) and the stage of speciation (complete or incomplete), both of which in fact represent continua [21,24]. Therefore, it may be beneficial to focus on a more mechanistic approach to identifying processes of divergence and speciation [12], by assessing processes that may lead to reproductive isolation in the absence of physical dispersal barriers.

*Seriatopora hystrix *is a scleractinian coral with a brooding reproductive strategy (i.e., eggs develop in the maternal colony and are released as larvae) and is common across a wide range of reef environments throughout the Indo-Pacific [25]. Larval dispersal of *S. hystrix *is strongly localized (with the majority of larvae settling within 100 m of the natal colony [26]), and *Symbiodinium *are vertically acquired (i.e., offspring obtain their symbionts from the maternal colony). In a previous study, we observed strong genetic partitioning of *S. hystrix *and its algal endosymbionts (*Symbiodinium*) across reef habitats (*'Back Reef'*, *'Upper Slope' *and *'Deep Slope'*), spanning a depth range of ~30 m at three locations on the far northern Great Barrier Reef (GBR) [27]. Mitochondrial and nuclear loci of the coral host animal indicated the occurrence of little to no gene flow between habitat-associated populations. However, the extent and nature of reproductive isolation (endogenous and/or exogenous reproductive barriers) remains unclear and difficult to assess due to the extreme difficulty of performing cross-fertilisation experiments in brooding corals. In contrast to the sharp differentiation across adjacent habitats, genetic similarity (both for host and symbiont) was observed between the same habitat types at different geographic locations [27], implicating environmentally-based divergent selection as the major driver of the observed differentiation. Nonetheless, the underlying ecological mechanisms shaping the habitat partitioning remain unknown. Many abiotic and biotic parameters vary over depth and therefore selective regimes can vary greatly among habitats [28]. Light presents an important selective factor due to the strong dependence of corals on light for energy requirements [29] and is regarded as a key factor in the depth-zonation of scleractinian coral species [30,31]. Both the coral host and associated *Symbiodinium *can exhibit adaptive traits to particular light environments and, thus, the ability of *S. hystrix *to thrive over large depth ranges may in part be due to the association with different types of *Symbiodinium *[27,32]. However, it is unclear whether the host-symbiont associations in *S. hystrix *are flexible or whether they are coupled taxonomic units with natural selection acting on the level of the holobiont (coral host and associated microbial symbionts, including *Symbiodinium*).

Here, we address whether the observed divergence of *S. hystrix *ecotypes (i.e., host-symbiont associations) results from divergent selection across environments and whether photo-physiology is likely to be a trait under selection. We performed a 14-month reciprocal transplantation experiment across the same three reef habitats (*'Back Reef'*, *'Upper Slope' *and *'Deep Slope'*) as in Bongaerts et al. [27] and used molecular and photo-physiological analyses to test whether: (1) differential selective regimes exist across habitats that can account for the observed partitioning (divergent selection), (2) host-symbiont associations can change in response to a change in environment (host-symbiont recombination), (3) selection has specifically led to fixed photo-physiological differences between ecotypes (adaptation), and (4) ecotypes can photo-acclimatise to conditions outside of their natural distribution range (phenotypic plasticity).

## Methods

### Experimental design

**Figure 1:**
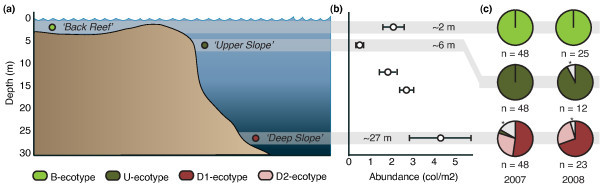
**Sampled habitats, abundances and host/symbiont diversity**. (*a*) The different habitats sampled at Yonge Reef. (*b*) Natural abundances of *Seriatopora hystrix *over depth. Error bars indicate ± SEM. (*c*) Host genotypic diversity of *S. hystrix *populations in the three habitats on Yonge Reef in 2007 [27] and 2008. Colours indicate host and symbiont genotypes as defined in Figure 3. Asterisk and grey fill indicates other genotypes occurring in low abundance.

A reciprocal transplantation experiment of the coral *Seriatopora hystrix *was carried out at Yonge Reef (14°36'59.9"S; 145°38' 11.1"E), located along the continental shelf edge on the far northern GBR from the 10^th ^of December 2008 until the 7^th ^of February 2010. Corals were cross-transplanted from/to three different habitats: the *'Back Reef' *(2 m depth ± 1 m), *'Upper Slope' *(6 m depth ± 1 m) and *'Deep Slope' *(27 m depth ± 2 m) (Figure 1a), which are identical to those sampled in Bongaerts et al. [27]. Temperatures in the three habitats were monitored during the transplantation experiment (~14 months) and the 12 months preceding the experiment using temperature sensors (HOBO U22 Water Temp Pro v2) logging at a 15 min interval. Light conditions in the three habitats were momentarily assessed on the 6^th ^of February 2010 between 10:10 and 11:40 using two 2π PAR loggers (Odyssey, New Zealand). Abundances of *S. hystrix *across the depth range were estimated by counting the number of colonies along 1 m wide belt-transects (7.5-10 m long) at each of the following depths: 2 m, 6 m, 12 m, 15 m and 27 m.

Fragments of *S. hystrix *colonies were collected from the three habitats (*'Back Reef'*, n = 25; *'Upper Slope'*, n = 13; *'Deep Slope'*, n = 24) and transported back to aquaria with flowing seawater at the Lizard Island Research Station (LIRS). The corals were maintained under shaded conditions at all times using neutral density screens. After sunset, fragments were split into three nubbins, which were then attached to three different transplantation racks (one for each habitat). One small piece was preserved in 20% DMSO for genotyping purposes. Transplant racks were redeployed to the three habitats within 26 h (± 2 h) of collection (11^th ^of December 2008). After nearly 14 months (on the 6^th ^of February 2010), the racks were recovered and transported in aquarium bins back to the LIRS (within 1-3 h of collection), where they were kept in flowing seawater under shaded conditions. Survival of fragments was assessed using the following categories: healthy (colony consisted mostly of living tissue), mostly dead (some living tissue, but extensive partial mortality with large parts of the skeleton covered in algae), and dead (no living coral tissue present on the skeleton). Missing fragments were excluded from the analyses (reducing the total number of transplanted fragments in each of the habitats). Turf algae and symbiotic crustaceans were removed from the nubbins and a small piece of the nubbin was preserved in 20% DMSO preservation buffer for genotyping purposes. After that, coral fragments were placed in an aquarium with flowing seawater under shaded conditions for several hours until they were subjected to respirometry measurements.

### Genetic characterisation

After DNA extraction (following Wilson et al. [33]), the putative control region (and parts of the adjacent *atp6 *and *nad4 *regions) of the coral host mitochondrial DNA (mtDNA) was amplified using the F18 and R17 primer pair [34] for the colonies used in the transplantation experiment (n = 62). For the surviving transplanted fragments (n = 48), a partial fragment of the putative control region (containing most variability) was amplified using the SerCtl-F1 and SerCtl-R1 primer pair [27]. PCR amplifications were performed using the cycling conditions as described in van Oppen et al. [35].

For the associated *Symbiodinium*, the internal transcribed spacer (ITS2) region of the rDNA was amplified for all samples at the beginning (n = 62) and end (n = 48) of the transplantation experiment, using the *Symbiodinium*-specific primers (ITSintfor2 and ITS2Clamp) and cycling conditions as described in Bongaerts et al. [27]. Amplified ITS2 fragments were run on a CBScientific Denaturing Gradient Gel Electrophoresis (DGGE) system using the conditions described in Sampayo et al. [36]. Representative, dominant bands of each characteristic profile were excised, eluted overnight in dH_2_O and re-amplified using the non-GC primers [37,38].

PCR reactions were purified using ExoSap and sequenced using both forward and reverse primers (ABI BigDye Terminator Chemistry, Australian Genome Research Facility). All chromatograms were aligned using Codoncode Aligner (version 3.7), checked manually and blasted on Genbank (http://www.ncbi.nlm.nih.gov/BLAST/). For the host sequences, an unrooted sequence network was generated, using the program TCS (version 1.21) [39], treating gaps as a fifth character state. For the *Symbiodinium *sequences, maximum parsimony analysis was run in PAUP* 4.0b10 [40] under the delayed transition option and using indels as a fifth character state, from which an unrooted sequence network was generated.

### Physiological characterisation

Respirometry assays were carried out in 82 cm^3 ^acrylic chambers filled with filtered seawater (0.45 μm) and fitted with a magnetic stir-bar on the bottom (to ensure consistent flow and simulate natural convection). The chambers were maintained at approximately 28°C (i.e., *in situ *temperature measured on the day of collection) using a temperature-controlled waterbath. Respiration and light-saturated rate of net photosynthesis (P_net_) were measured by recording changes in O_2 _concentrations using oxygen probes (Oxy4 v2 sensor, PreSens, Germany). Using an optical filter (Lee Filters #120, Hants, UK) in combination with metal halide lamps, fragments were exposed to two irradiances of different spectral composition that matched the conditions in the '*Deep Slope*' (blue light spectrum; 120 μmol photons m^-2 ^s^-1^) and '*Upper Slope*' (full light spectrum; 1200 μmol photons m^-2 ^s^-1^) habitats. Oxygen flux was determined during daylight hours for 15 min under blue light, 15 min under full light, followed by 20 min in the dark. Light enhanced dark respiration (LEDR) rates were determined in the 10 min immediately following lights out, whereas dark respiration (DR) rates were determined in the final 10 min. After completion of the respirometry assays, the fragments were maintained in the dark and transferred to liquid nitrogen, then stored at -80°C until further processing for pigment analyses.

Coral surface area was determined by double dipping the coral fragment in melted paraffin [41] and coral volume was determined by measuring the volume of water displaced by the fragment immersed in the 82 cm^3 ^respirometry chambers. After airbrushing the frozen tissue, the dinoflagellate endosymbionts were separated following Dove et al. [42]. Cell densities of *Symbiodinium *were determined by counting 6 independent subsamples using a Sedgewick rafter cell 550 (ProSciTech s8050, Kirwin, Queensland, Australia). *Symbiodinium *pigments were analysed using high performance liquid chromatography as described in Dove et al. [43]. Pigments were separated following the method of Zapata et al. [44], and quantified via co-elution with pigment standards for chlorophyll *a*, chlorophyll *c*_*2*_, ß-carotene, diadinoxanthin and diatoxanthin (the latter two forming the dinoflagellate xanthophyll pool).

### Statistical analyses

Differences in holobiont genotypic diversity between the two sampling years (2007 and 2008) in each of the three habitats were assessed using a nested analysis of similarity (two-way ANOSIM; habitat nested within year) to check for temporal consistency of the habitat partitioning across sampling years. Differences in the abundances of *S. hystrix *in the three habitats were log-transformed and assessed using analysis of variance (one-way ANOVA). The effect of source and destination on survival was assessed using 2 × 3 contingency tables with destination habitat (*'Back Reef'*, *'Upper Slope' *and *'Deep Slope'*) as explanatory values and survival ('dead' and alive') as response variables. Two separate sets of ANOVA were performed for the physiological data, due to the lack of survival of fragments from the '*Deep Slope*' in either of the shallow habitats. The effects of both source (i.e., the habitat the fragments were collected from) and destination (i.e., the habitat the fragments were transplanted to) were assessed using two-way ANOVA for transplants originating from the *'Back Reef' *and *'Upper Slope' *habitats. One-way ANOVAs were used to assess the effect of source habitat for fragments transplanted to the '*Deep Slope*' habitat. All statistical analyses were done using STATISTICA 7.0 (Statsoft Inc.) and PRIMER 6 (PRIMER-E Ltd.).

## Results

### Abundances, environmental conditions, and host-symbiont genotypic diversity

Across the three sampled habitats (Figure 1a), abundances of *S. hystrix *(Figure 1b) were significantly lower in the *'Upper Slope' *habitat (0.5 ± 0.06 colonies/m^2^) than in the '*Deep Slope' *(p < 0.005; Fisher's LSD post-hoc test) and *'Back Reef' *(p < 0.05; Fisher's LSD post-hoc test) habitats. The '*Deep Slope' *habitat had the highest recorded abundances with 4.3 colonies/m^2 ^(± 1.5).

**Figure 2:**
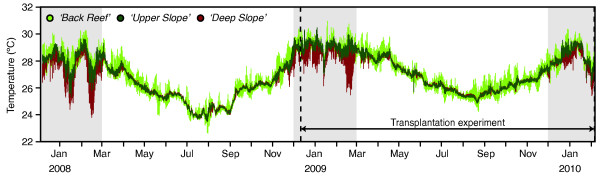
**Temperature patterns in the three different habitats**. Temperature records before and during the transplantation experiment. Warmest months (Dec-Mar) are indicated in grey, which is when the cold-water influxes occur in the '*Deep Slope*' habitat.

Temperatures recorded over 26 months at the site of transplantation exhibited a strong seasonal fluctuation, with monthly means varying from 27.4 - 29.1°C during the warmest summer months (January/February) to 24.0 - 25.8°C in the coldest winter months (July/August) (Figure 2). Monthly temperature averages were similar over depth, except for the summer months when strong temperature fluctuations (typically 0.5 - 2°C over the course of 15 minutes) occurred in the '*Deep Slope*' habitat due to influxes of colder, deep oceanic water. Furthermore, compared to the '*Upper Slope*' habitat (~6 m), the *'Back Reef' *habitat (~2 m) showed greater daily temperature variability throughout the year, with departures of up to 2°C from the daily mean occurring in relation to the tides.

Incident irradiances measured at the different habitats between 10:10-11:40 AM, on a cloudless day, gave values of 1339 μM Quanta m^-2^s^-1 ^for the '*Back Reef' *habitat, 693 μM Quanta m^-2^s^-1 ^for the '*Upper Slope*' habitat, and 136 μM Quanta m^-2^s^-1 ^for '*Deep Slope*' habitat, with an extrapolated Kd [PAR] of 0.085. These values corresponded well with a Kd [PAR] of 0.084 measured during the summer solstice period on the adjacent Ribbon Reefs (Veal et al. unpublished data). Thus, even though Kd values and surface irradiances fluctuated on a daily and seasonal basis, the proportion of surface irradiance available in the '*Deep Slope' *habitat was roughly 10 times lower than in the shallower habitats, whereas the incident irradiance in the *'Back Reef' *was about twice that available in the *'Upper Slope' *habitat.

**Figure 3:**
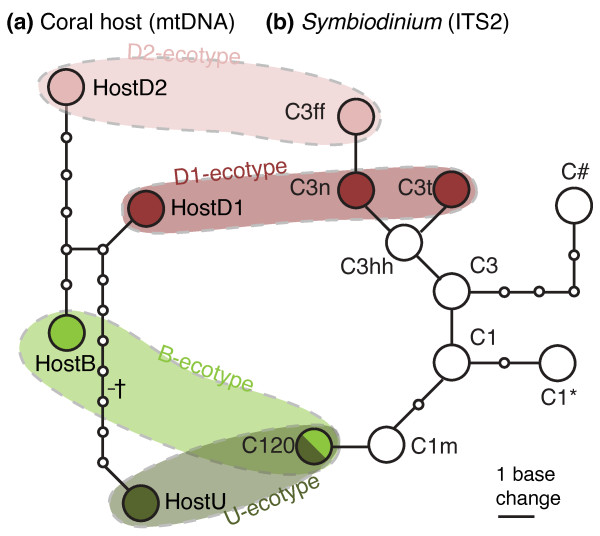
**Haplotype networks of *Seriatopora hystrix *(mtDNA) and the associated *Symbiodinium *(ITS2)**. Unrooted haplotype networks of (*a*) *Seriatopora hystrix *(mtDNA) and (*b*) the associated *Symbiodinium *(ITS2). Colours indicate host and symbiont genotypes that together constitute the four different ecotypes: B-ecotype, U-ecotype, D1-ecotype and D2-ecotype, which are grouped together by the shaded ovals. Dagger (†) indicates a 51bp tandem repeat that was excluded from the analysis.

Analyses of the putative control region (1322 - 1372 bp) from host mitochondrial DNA and the ITS2 region (284 - 287 bp) of *Symbiodinium *rDNA revealed 4 mtDNA haplotypes (HostB, HostU, HostD1 and HostD2) and 3 dominant *Symbiodinium *profiles respectively (C120, C3n-t and C3-ff), which are identical to genotypes described in Bongaerts et al. [27] and van Oppen et al. [35]. Host and symbiont genotypes also exhibited a near-identical pattern of habitat zonation (Figure 1c) and host-symbiont association (Figure 3) to that observed in 2007: the B-ecotype (HostB in association with C120) was dominant in the *'Back Reef'*, the U-ecotype (HostU in association with C120) in the *'Upper Slope*,*' *and the D1-ecotype (HostD1 in association with C3n-t) and D2-ecotype (HostD2 in association C3-ff) in the '*Deep Slope' *habitat (Figure 1c). As such, holobiont genotypic diversity was significantly different (two-way ANOSIM; habitat nested within year) between habitats (R = 0.769; p = 0.001) but not between years (2007 vs. 2008). Two additional host-symbiont genotype associations were observed that occurred only once: one colony with the HostD2 haplotype was found in association with *Symbiodinium *C3n-t and another colony with a HostU haplotype was observed to host a mix between *Symbiodinium *C120 and C1m (Figure 3b). Additionally, several colonies exhibited profiles designated as C120, but with several faint extra bands in the profile. On several occasions, sequences of these bands were successfully recovered and these represented a novel ITS2 sequence: C1* (Genbank Accession Number JF320827) and an unnamed sequence previously reported in *S. hystrix *colonies in North West Australia: C# (Genbank Accession Number JF298202; [35]).

### Transplant survival and symbiont stability

**Figure 4:**
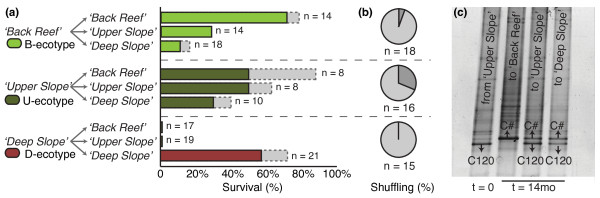
**Survival and symbiont shuffling of transplanted fragments**. (*a*) Survival rates one year after transplantation. Coloured bars refer to healthy fragments, whereas the grey fraction refers to transplanted fragments that still had some living tissue, but were mostly dead. Sample sizes (n) refer to the total number of fragments (alive or dead) present on the rack after 14 months (missing fragments were excluded). (*b*) Incidence of symbiont shuffling (i.e., change in the abundance of already present *Symbiodinium*). Dark grey indicates shuffling, whereas light grey indicates no change in symbiont community. Note that in none of the corals, new *Symbiodinium *types were acquired (i.e., symbiont "switching"). (*c*) Example of changes in *Symbiodinium *profile for a transplanted colony originating from the '*Upper *Slope' habitat.

Overall survival of transplanted fragments (n = 129) after the 14 month transplantation period was 38%. In all cases, genotyping of the surviving fragments, using a variable subsection of the putative control region (557-608 bp), reconfirmed the genetic identity as determined at the beginning of the experiment. Survival of fragments (excluding fragments with extensive partial mortality) originating from the *'Back Reef' *(B-ecotype) was significantly dependent on destination (*X*^2 ^= 14.1; p = 0.001) and highest (71%) when transplanted back to the same habitat, in comparison to when transplanted to either the *'Upper Slope' *(29%) or *'Deep Slope' *habitat (11%) (Figure 4a). Fragments originating from the *'Deep Slope' *(D1 and D2 ecotypes) only survived when transplanted to the same habitat (71%) and never survived in the shallower habitats (n = 36) (*X*^2 ^= 30.9; p < 0.001). No apparent differences in survival rates were observed for the HostD1 and HostD2 genotypes. Survival of fragments originating from the *'Upper Slope' *habitat was not significantly dependent on destination (*X*^2 ^= 2.90; p = 0.234), however highest survival rates were recorded in the *'Back Reef' *(50%) and *'Upper Slope' *habitat (50%), with lower survival rates when transplanted to the *'Deep Slope' *habitat (30%).

Regardless of transplantation origin and destination, the majority of surviving fragments (95%) harboured a symbiont profile identical to that before transplantation (Figure 4b). Four colonies, one originating from the *'Back Reef' *and three originating from the *'Upper Slope' *habitat, were observed to exhibit changes in *Symbiodinium *profile after transplantation. In three colonies, the C120 profile changed after transplantation to either C# or a mix of C120 and C# (Figure 4c). The symbiont profile of a fourth colony (from the *'Upper Slope' *habitat), changed from a C120 profile to a mix of C120 and C1*. Although the ITS2-DGGE technique is limited in its ability to detect *Symbiodinium *types present in low abundances and to quantify relative abundances, the new dominant bands observed after transplantation were already observed as a very faint band in the original sample, indicating that *Symbiodinium *types were not newly acquired.

### Photo-physiological measurements

**Figure 5:**
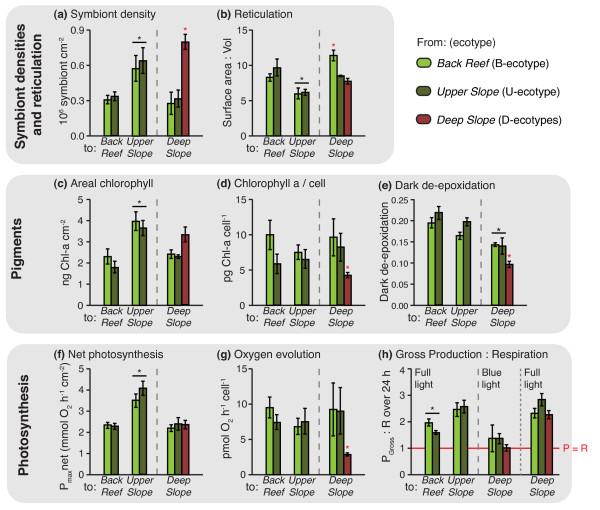
**Photo-physiological responses after transplantation**. (*a*) Symbiont density, (*b*) Reticulation, (c) Areal cholorphyll *a*, (*d*) Chlorophyll *a*/cell, (*e*) Dark de-epoxidation, (*f*) Net photosynthesis (in full light), (*g*) Oxygen evolution/cell, (*h*) Gross production to respiration over 24 h. Colours indicate source habitat and x-axis indicates destination habitat. Error bars indicate ± SEM. Asterisks indicate significant differences in the one-factorial (red asterisk above individual bar) and two-factorial (black asterisk with line across two bars) analyses.

Given that no fragments from the '*Deep Slope*' survived in either of the shallow habitats ('*Upper Slope*' or '*Back Reef*') (Figure 4a), the photo-physiological data were investigated using (1) two-factorial analyses to look at the effects of both source and destination on fragments originating from the shallow habitats (B- and U-ecotypes) and relocated to all three habitats, and (2) one-factorial analyses to look at the effect of source habitat (B-, U- and D-ecotypes) on fragments transplanted to the '*Deep Slope*' habitat (Figure 5; Table 1). In the two-factorial analyses, no significant differences between B- and U-ecotypes (source) were observed in any of the measured traits (Table 1), indicating that these two ecotypes (that also harbour the same symbiont C120), exhibited similar photosynthetic responses.

**Table 1:** ANOVA results of the photo-physiological responses

	two-factorial ANOVA				one-factorial ANOVA		
	source	destination		SNK (destination)	source		SNK (source)
*Symbiont densities and reticulation*							
Symbiont density	n.s.	p < 0.0004	F(2,28) = 10.67	U > B = D*	p < 0.002	F(2,15) = 9.7918	D > B = U
Reticulation	n.s.	p < 0.001	F(2,28) = 9.01	U < B = D	p < 0.009	F(2,15) = 6.64	B > D = U
							
*Pigments*							
Chlorophyll *a*/cell	n.s.	n.s.			p < 0.002	F(2,15) = 9.8706	D < B = U
Areal chlorophyll	n.s.	p < 0.0004	F(2,28) = 10.13	U > B = D*	n.s.		
Dark-adapted XDE	n.s.	p < 0.005	F(2,28) = 6.65	D < B = U	p < 0.022	F(2,15) = 5.0157	D < B = U
Chlorophyll *c *: *a*	n.s.	n.s.			n.s.		
Xantophyll: Chlorophyll *a*	n.s.	n.s.			n.s.		
ß-Carotene: Chlorophyll	n.s.	p < 0.008	F(2,28) = 5.86	D < U =(<) B	n.s.		
							
*Photosynthesis Photosynthesis*							
Oxygen evolution per cell	n.s.	n.s.			p < 0.0023	F(2,14) = 9.7749	D < B = U*
Net Photosynthesis (P)	n.s.	p < 0.000006	F(2,28) = 25.73	U > B = D	n.s.		
Gross P: R over 24 h^#^	n.s.	p < 0.0001	F(2,28) = 13.05	B < U = D	n.s.		
Blue: full light	n.s.	n.s.			n.s.		

SNK = Student-Newman-Keuls test; XDE = Xanthophyll de-epoxidation; ^#^full light for destination DE; * ANOVA assumptions not met, but p << 0.01

The two-factorial analyses test for source and destination effects of transplanted fragments originating from the '*Back Reef*' (B) and '*Upper Slope*' (U) habitats (note that source effects were never significant). The one-factorial analyses test for source effects of fragments originating from the '*Back Reef*' (B), '*Upper Slope*' (U) and '*Deep Slope*' (D) habitats transplanted to the '*Deep Slope*' habitat.

Symbiont densities (i.e., number of *Symbiodinium *cells per surface area) and reticulation (i.e., ratio of surface area to volume) differed significantly depending on destination (two-factorial analyses; Table 1), indicating a certain degree of environment-induced plasticity in these traits. Significantly higher symbiont densities were observed for shallow ecotypes transplanted to the '*Upper Slope*' habitat (Figure 5a), as well as lower reticulation (i.e., more compacted growth forms) (Figure 5b). When comparing all three ecotypes after transplantation to the '*Deep Slope*' habitat (one-factorial analyses; Table 1), D-ecotypes were found to exhibit the highest symbiont densities compared to the other ecotypes (Figure 5a). Additionally, reticulation was highest for the B-ecotype (Figure 5b), which represented the only observed (significant) trait differentiation between the B and U-ecotype.

Several pigment traits differed significantly depending on destination (two-factorial analyses; Table 1), with significantly higher levels of areal chlorophyll (i.e., chlorophyll per surface area) for fragments transplanted to the '*Upper Slope*' habitat (Figure 5c). Additionally, dark-adapted xantophyll de-epoxidation (i.e., the ability to maintain thylakoid potential in the absence of light) and the concentration of β-carotene (i.e., pigment involved in photo-protective mechanisms) were significantly lower for fragments transplanted to the '*Deep Slope*' habitat (Figure 5e, Table 1). Other pigment traits, such as chlorophyll *a *per cell, chlorophyll *c *: *a *ratio, and the concentrations of xantophyll pigments (sum of Diatoxanthin (Dt) and Diadinoxanthin (Dd) per chlorophyll *a*) did not vary significantly depending on destination habitat (Figure 5d; Table 1). When assessing source effects for fragments transplanted to the '*Deep Slope*' habitat (one-factorial analyses; Table 1), D-ecotypes exhibited the lowest concentrations of chlorophyll *a *per cell and xantophyll dark de-epoxidation (Figure 5d, e), with no significant differences between other pigment traits. In fact, the low symbiont densities with high chlorophyll per cell (as observed for B- and U-ecotypes) lead to similar levels of areal chlorophyll to that of the D-ecotypes, that exhibited high symbiont densities with low chlorophyll per cell (Figure 5a,c,d).

Net maximum photosynthesis (P_net _max) was significantly higher for B- and U-ecotypes transplanted to the '*Upper Slope*' habitat, whereas net photosynthesis was similar for fragments transplanted to '*Back Reef*' and '*Upper Slope*' habitats (Figure 5f). The rate of oxygen evolution per cell also did not differ significantly across the different habitats (two-factorial analyses; Table 1). However, when assessing source effects for fragments transplanted to the '*Deep Slope*' habitat (one-factorial analyses; Table 1), D-ecotypes exhibited significantly lower rates of oxygen evolution per cell (i.e., photosynthetic efficiency of individual symbionts) (Figure 5g). Coral fragments are potentially autotrophic when the ratio of gross production (P_gross_) to respiration (R) is greater than 1 over the long-term [45], with R including carbon respired as a result of maintaining and/or replacing existing tissue, and carbon respired as a result of depositing new tissue (growth) [46]. Under full light, shallow ecotypes transplanted to the '*Back Reef*' had significantly lower P_gross_: R than those transplanted to the '*Upper Slope*' or the '*Deep Slope*' (two-factorial analyses; Table 1), but in all cases the P_gross_: R was greater than 1 (Figure 5h). However, calculations of P_gross_: R using blue light for fragments transplanted to the '*Deep Slope*' habitat, indicated that fragments irrespective of source habitat may only be borderline phototrophs in the deep. Even fragments of the D-ecotype relocated back to the '*Deep Slope*' were on average evolving O_2 _at only 45% of P_net _max under blue light (i.e., mimicking natural deep-water light conditions) (Figure 5h).

## Discussion

We observed differential survival of ecotypes across habitats, no recombination between host and symbiont genotypes after transplantation, and photo-physiological differences between shallow and deep *S. hystrix *ecotypes. These observations confirm that the partitioning of these highly coupled host-symbiont associations across reef habitats reflects a process of adaptive divergence that is likely to be driven by environmental divergent selection.

### Differential survival of ecotypes across habitats

The genotypic composition of habitats at the beginning of the transplantation experiment in 2008 was nearly identical to that observed in 2007, with four dominant ecotypes occurring in association with particular reef habitats (Figure 1c). Each ecotype was only found in association with a single habitat type, although a few colonies of the dominant '*Upper Slope*' ecotype were observed in the '*Deep Slope*' habitat in 2007 (~6%) [27]. The survival rates of transplanted fragments reflected this zonation, as survival rates were generally highest when transplanted back to the same habitat (Figure 4a). This pattern was most apparent for the '*Back Reef*' and '*Deep Slope*' ecotypes, although the lack of survival of '*Deep Slope*' transplants in the shallow habitats may in part be the result of instant light stress after transplantation. Although the point in time at which mortality of these non-native transplants occurred is unknown, the sudden exposure to high-light conditions could potentially have resulted in oxidative stress and/or dissociation of the coral-*Symbiodinium *symbiosis [47,48]. A gradual change in the depth of transplants over a long period of time may have prevented complete mortality of the deep to shallow transplants [49], however this was logistically not feasible in the current study. The lack of significant differences in survival rates across habitats for the '*Upper Slope*' ecotype may reflect the slightly more opportunistic nature of this ecotype, as this ecotype is also observed in deeper water at low abundance (Figure 1c; Bongaerts et al. unpublished data). Overall, there is compelling evidence for selection against ecotypes outside their natural distribution range, indicating differential selective regimes across habitats.

Because selection pressures may vary depending on the life stage of a coral, small singular branches of *S. hystrix *were transplanted to mimic well-established juvenile corals (1-2 years old; [50,51]) and exclude selective effects based on gross colony morphology. Nonetheless, by using branches of adults, survival rates may be affected by effects of long-term acclimatisation or developmental canalisation (i.e., fixation of phenotypic traits during early life stages; [52]). Developmental canalisation of corals is poorly understood, and is a common confounding factor in transplantation experiments with anthozoans that is difficult to overcome [53]. However, developmental canalisation probably occurs even before settlement in brooding corals as larval development happens per definition within the maternal colony and *Symbiodinium *are usually vertically acquired (i.e., through maternal transmission). Thus, developmental canalisation may actually play an important additional role in reinforcing selective recruitment. Pre-settlement processes (e.g., active habitat selection) are believed to drive interspecific differences between distribution ranges in corals [54,55], and although evidence is lacking, such processes may similarly reinforce depth partitioning at an intraspecific level [56]. Despite the potential contribution of developmental canalisation and/or pre-settlement processes, our results demonstrate that differential selection pressures across habitats are at least partially responsible for the observed habitat partitioning of ecotypes through post-settlement selection.

### Stability of host-symbiont associations

The different host-symbiont assemblages (i.e., the four ecotypes) appear to represent stable associations, as identical combinations of host and symbiont genotypes were observed in the surveys of 2007 and 2008 (Figure 1c) and because the vast majority of transplanted fragments (95%) did not exhibit any change in *Symbiodinium *profile over time (Figure 4b). Although the four different host mtDNA lineages (Figure 3) have been found in association with various *Symbiodinium *types [27,35], host-symbiont associations are specific in that there is a clear separation between shallow (e.g. 'HostB', 'HostU', 'C120') and deep genotypes (e.g. 'HostD1', 'HostD2', 'C3n-t', 'C3-ff') (Figure 3). In the few fragments for which symbiont profiles did vary after transplantation (n = 6), the new dominant bands were already present as a faint band in the original sample and therefore represent examples of symbiont "shuffling" [57-59]. Thus, no definitive host-symbiont recombination (symbiont "switching") between shallow and deep genotypes was observed. The stability of host-*Symbiodinium *associations over time and after a change in environment (transplantation) reinforces the status of these host-symbiont assemblages as highly specific, coupled taxonomic units in *Seriatopora hystrix*. It is likely that the vertical symbiont acquisition strategy plays an important role in maintaining this tight association [38,60,61]. As such, natural selection operates at the level of the holobiont (host and symbiont), resulting in co-diversification [62,63].

### Physiological differences between shallow and deep ecotypes

Differences in photo-physiological responses were observed between shallow and deep ecotypes after 14 months in the same habitat (Figure 5). Although all ecotypes exhibited similar photosynthetic performance by surface area and had similar areal chlorophyll concentrations after transplantation to the '*Deep Slope*' habitat, the photosynthetic efficiency of individual symbionts (rate of oxygen evolution per symbiont cell) was significantly higher for the shallow ecotypes (B- and U-ecotypes). The differences in photosynthetic efficiency reflect two opposing strategies: shallow ecotypes had low *Symbiodinium *densities with high chlorophyll concentrations per symbiont cell, whereas deep ecotypes had high *Symbiodinium *densities with low chlorophyll concentrations per symbiont cell. Additionally, shallow ecotypes showed an increased ability to quench energy from excessive light through xanthophyll de-epoxidation. Although an effect of environment on xanthophyll de-epoxidation was also detected (plasticity), there were significant differences between shallow and deep ecotypes when transplanted to the same habitat ('*Deep Slope*').

**Figure 6:**
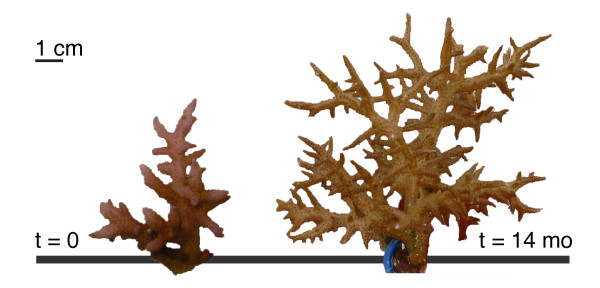
**Growth example of transplanted fragment**. Example of growth during the transplantation period (14 months) of a fragment transplanted back to the '*Deep Slope*' habitat.

Deep ecotypes did not appear to be particularly well adapted to the low-light conditions in their native '*Deep Slope*' habitat. The net photosynthetic performance of deep ecotypes was similar to that of shallow ecotypes transplanted to the '*Deep Slope*' habitat (Figure 5f), and photosynthetic use of blue, low intensity photons was relatively inefficient. In fact, the ratio of gross photosynthesis to respiration (in blue light) hovered around 1 for all ecotypes when transplanted to the '*Deep Slope*' habitat (Figure 5h). This indicates, that even the D-ecotypes may only be borderline phototrophic in their native habitat, and may not be well supported by the photosynthetic capacity of their zooxanthellae. However, abundances of *S. hystrix *(mostly comprising of the D1-ecotype) in the '*Deep Slope*' habitat were very high compared to shallower depths (Figure 1b), indicating good habitat quality [64]. Furthermore, although growth and health were not quantitatively assessed, D-ecotype fragments transplanted back to the '*Deep Slope' *in several cases exhibited extensive growth (Figure 6) in comparison to shallow ecotypes.

**Figure 7:**
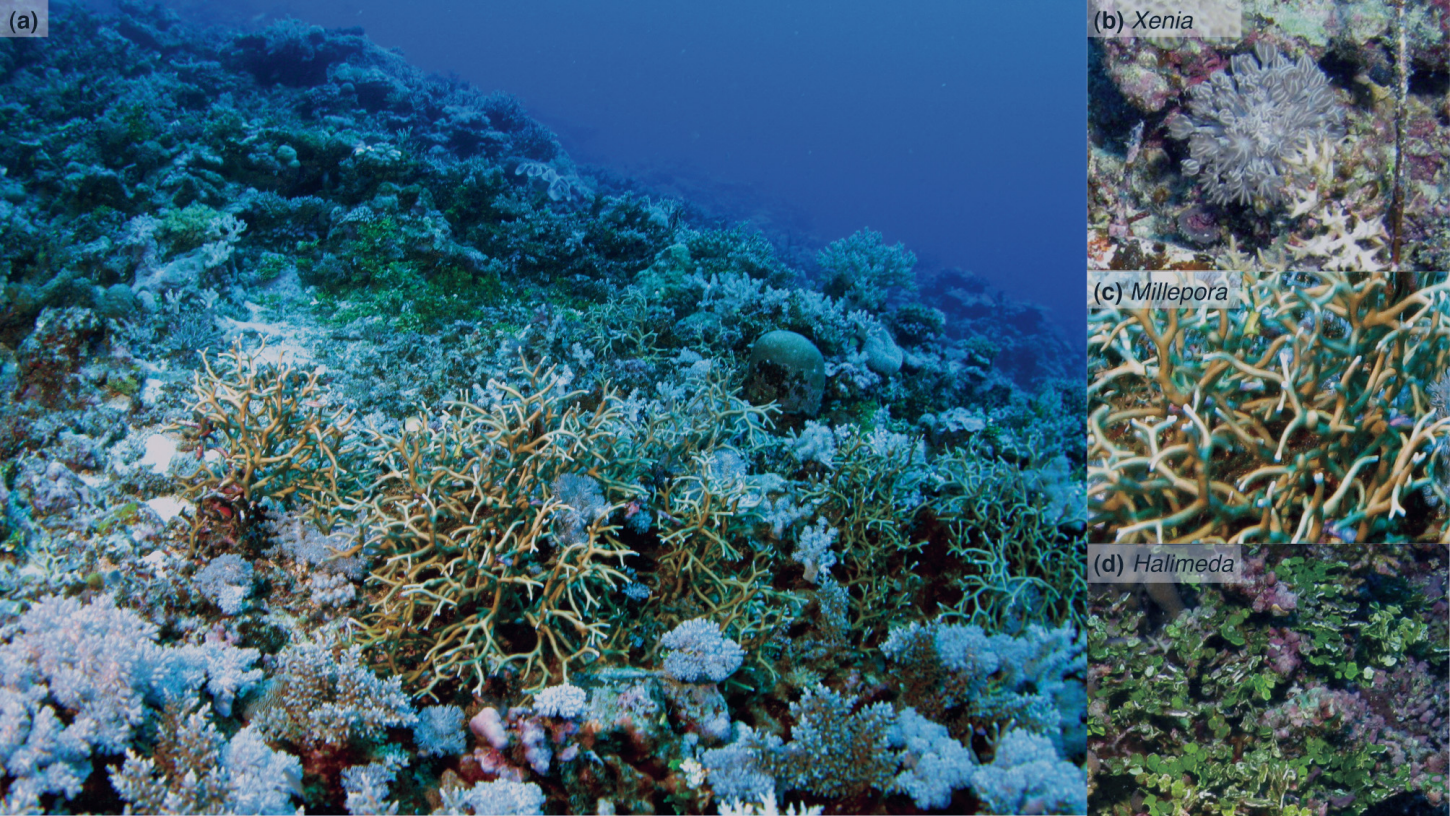
**'*Deep Slope*' habitat**. (*a*) Photo of '*Deep Slope*' habitat, showing the abundance of: *Xenia *and *Millepora *(both in the front), and *Halimeda *(in the back). (*b*) *Xenia*. (*c*) *Millepora*. (*d*) *Halimeda*.

The disjunction between photosynthetic performance and ecological success (in terms of growth and abundance) points to an increased ability of deep ecotypes to exploit energy sources other than light [65]. Increased symbiont densities (as observed in the D-ecotypes) is a common response in well-fed corals that are kept under low-light conditions, and inter-specific differences in cell-specific densities (i.e., number of zooxanthellae contained in each individual host cell) have been related to different feeding capacities of various coral species (reviewed in [66]). Also, in the absence of enhanced feeding, the "phototrophic response" to lower light conditions would be an increase in antennae (reflected as increased chlorophyll per symbiont cell) to enhance capture of the available light [67], which was not observed. The vast abundance of *Halimeda*, *Xenia *and *Millepora *spp. in the '*Deep Slope*' habitat (Figure 7) and at mesophotic depths (>30 m), combined with the cold-water influxes observed in summer (Figure 2), are important indicators of increased nutrient and plankton availability in the deeper habitat [68,69]. Thus, it is likely that deep ecotypes have an increased ability to take advantage of heterotrophic resources, rather than being adapted to low-light conditions.

### Similar photo-physiological plasticity in shallow ecotypes

The two shallow-water, mitochondrially defined ecotypes were partitioned across the '*Back Reef*' and '*Upper Slope*' habitats (Figure 1c). Yet, they associated with the same *Symbiodinium *type (C120) and exhibited a similar degree of photo-physiological plasticity, allowing them to acclimatise to light conditions in either habitat (Figure 5). Similar environment-related responses in xantophyll de-epoxidation, β-Carotene to chlorophyll ratio, and amount of chlorophyll per surface area were observed for both ecotypes. However, chlorophyll concentrations per cell did not vary significantly when transplanted to different environments and may therefore represent a fixed trait. The lack of significant differences in photo-physiological responses between the two shallow ecotypes indicates that the strong partitioning (Figure 1c) and differential mortality (Figure 4a) of the B- and U- ecotypes across the '*Back Reef*' and *'Upper Slope*' habitats must be due to other adaptive traits, which were not detectable through photo-physiological proxies. For example, in addition to high irradiance levels, the '*Back Reef*' habitat exhibits strong temperature fluctuations with departures of up to 2°C above the temperatures in the '*Upper Slope*' habitat (Figure 2), and the '*Back Reef*' population (B-ecotype) may therefore have a greater capacity to tolerate temperature fluctuations. In American Samoa, back- and fore-reef populations of *Porites lobata *were found to be genetically different and exhibited different levels of biomarker response (ubiquitin-conjugated proteins), potentially related to the different thermal conditions in these habitats [70]. Furthermore, the '*Upper Slope*' habitat is located just below the reef crest and experiences strong wave action, which is known to act as an important selective force on coral morphology. Some morphological plasticity in reticulation was observed for both shallow ecotypes after transplantation (Figure 5d), which may reflect morphological acclimatisation to the high-energy environment in the '*Upper Slope*' habitat. But, differences in gross morphology (although not quantified) were also observed across shallow habitats (with more compact colony shapes and thicker branches in the '*Upper Slope*' habitat), which may have a genetic basis (e.g. [71,72]).

## Conclusions

In this study, we demonstrate that the previously described genetic partitioning of *S. hystrix *across depth-related habitats (rather than geographic locations) reflects a process of adaptive divergence along a depth gradient [27]. Divergent selection appears to have acted on the taxonomic unit of the coral holobiont (i.e., coral host and associated *Symbiodinium*), leading to adaptive divergence of host-symbiont pairs across reef habitats.

Divergent selection across habitats constitutes an ecological barrier impeding migration between habitats and probably played an important role in reducing gene flow between populations along the depth gradient. Particularly, given the strongly localised dispersal of *S. hystrix *[26], the selection-driven partitioning of ecotypes across habitats would result in assortative mating (i.e., mating with neighbours), which may drive and/or strengthen the genetic divergence of the depth-associated populations. This scenario represents a mechanism of divergence-with-gene flow [73] that could explain how ecological barriers, rather than physical separation, may be responsible for the observed divergence in *S. hystrix*.

Despite the identification of a plausible mechanism for divergence in sympatry, it is impossible to rule out any contribution of historical allopatry to genetic divergence. Present-day selection appears to strongly reduce migration between habitats, however it is unclear whether the strength of this selective force was initially sufficient to drive divergence in the absence of other isolating mechanisms, or alternatively, whether reduced rates of migration played a role in instigating divergence. The different host lineages (i.e., mtDNA haplotypes) in this study form a monophyletic cluster that is distinct from the only other reported congeneric species on the GBR: *Seriatopora caliendrum *[27]. Three of the four dominant host mtDNA haplotypes have a widespread occurrence with overlapping distributions across the Indo-Pacific [34,35,74]). Although the present-day sympatric occurrence (geographically) of these sister lineages may be indicative of divergence in sympatry (*sensu *[19]), the widespread distribution could also be used to argue that divergence has occurred in allopatry followed by secondary contact. By this logic, it is impossible to falsify the contribution of past allopatry in divergence for species with wide ranging distributions, such as those that typify marine animals. Nevertheless, regardless of any past geographic context, our results demonstrate that ecologically-based divergent selection is a viable cause of divergence in scleractinian corals.

Although referred to in this study as "ecotypes", the exact point along the continuum of divergence (from differentiated populations to biological species) remains unknown for *S. hystrix*, and it is unclear whether additional isolating mechanisms beyond habitat isolation have evolved. The difficulty of performing cross-breeding experiments with brooding corals also hampers the ability to assess the fitness and/or viability of intermediate forms (i.e., hybrids), and therefore prevents independent assessment of exogenous and endogenous reproductive barriers. Although we observed natural selection against immigrants consistent with ecological (incipient) speciation [14], fitness tradeoffs could theoretically be the mere consequence of genetic drift [16,75]. The latter can only be ruled out by assessing whether hybrid fitness is also reduced by ecological mechanisms [14]. Thus, we are unable to empirically test whether ecologically-based divergent selection is the ultimate cause or a contributing factor in the divergence between *S. hystrix *ecotypes. Nonetheless, our findings corroborate similar observations in other marine taxa, such as tropical and temperate reef fish [76,77], snails [78] and limpets [79], which highlight the potential role of ecological barriers in species divergence (despite the extreme difficulty to falsify past allopatry in a marine context). Given the environmental heterogeneity encountered on coral reefs, processes of ecological adaptation may be an important contributor to diversification in scleractinian corals and their associated *Symbiodinium*.

## Authors' contributions

PB designed and conceived of the study, carried out the experiments, performed the genetic analyses, and drafted the manuscript. CR participated in genetic analyses, interpretation of data, and helped to draft the manuscript. KBH participated in the study design, data acquisition, and helped to draft the manuscript. MJHvO participated in genetic analyses, and helped to draft the manuscript. OHG participated in the study design and helped to draft the manuscript. SD participated in the data acquisition and analyses for the photo-physiological component of the study, and helped to draft the manuscript. All authors read and approved the final manuscript.
